# DNA Polymorphism of the *LOXL1* Promoter Region in Exfoliation Syndrome in Uygur Individuals in XinJiang, China

**DOI:** 10.1155/2022/9342635

**Published:** 2022-07-30

**Authors:** Yinu Ma, Mengting Yang, Xueyi Chen, Xianglong Yi

**Affiliations:** Department of Ophthalmology, The First Affiliated Hospital of Xinjiang Medical University, Urumqi 830011, Xinjiang Uygur Autonomous Region, China

## Abstract

**Purpose:**

On the basis of our previously reported work, the association of lysyl oxidase-like 1 (*LOXL1*) promoter region gene polymorphism with exfoliation syndrome (XFS) and exfoliation glaucoma (XFG) in Uygur individuals was examined.

**Methods:**

This was a case-control association trial. A total of 242 unrelated XFS/G and 310 control cases were assessed. The genotypes of 6 single nucleotide polymorphisms (SNPs) of the *LOXL1* promoter (rs4886761, rs4886467, rs4558370, rs4461027, rs16958477, and rs12914489) were examined via direct sequencing.

**Results:**

Each of the above SNPs had significant associations with XFS and XFG. The T allele of rs4886761 (OR (95% CI): 2.204 (1.711–2.838)), G of rs4886467 (OR (95% CI): 1.946 (1.513–2.503)), T of rs4461027 (OR (95% CI): 2.26 (1.773–2.881)), A of rs16958477 (OR (95% CI): 1.792 (1.399–2.297)), and G of rs12914489 (OR (95% CI): 1.103 (0.631–1.929)) independently predicted XFS/G. The genotypes TT and CC of rs4886761 (OR (95% CI): 5.655 (3.000–10.660) and 2.241 (1.473–3.408), respectively), TT and GG of rs4886467 (OR (95% CI): 4.026 (2.162–7.497) and 1.631 (1.08–2.463), respectively), CC and TT of rs4461027 (OR (95% CI): 5.245 (3.037–9.058) and 2.210 (1.37–3.564), respectively), CC and AA of rs16958477 (OR (95% CI): 3.530 (1.968–6.334) and 1.740 (1.145–2.646), respectively) also independently predicted XFS/G. The GGT and GTG haplotypes of rs12914489, rs4886467, and rs4558370 and TC and CT of rs4461027 and rs4886761 showed significant associations with XFS/G.

**Conclusions:**

These results confirmed LOXL1 as a susceptibility gene in XFS/XFG among Uygur individuals. The new SNPs of rs4886761, rs4886467, rs4461027, and rs16958477 polymorphisms are involved in the pathogenetic mechanism of XFS/G.

## 1. Introduction

Exfoliation syndrome (XFS) represents an age-associated, generalized pathology affecting the extracellular matrix (ECM), which features gradual accumulation of anomalous fibrillar materials in intraocular and extraocular structures [1, 2]. Exfoliation glaucoma (XFG) constitutes the commonest type of secondary open-angle glaucoma, occurring in individuals with XFS [3, 4]. In China, 3.3% of Han nationals [5], 5.1% of Kashi Uygur individuals [6], and 2.2% and 9.5% of Kuche Uygur people above 60 and 80 years, respectively, are affected [7].

SNP is based on the change of DNA sequence (polymorphism) caused by the variation of a nucleotide in the genome sequence. Since the molecular basis of all genetic polymorphisms is nucleotides, SNPs may reach the limit of the number of polymorphic sites in the human genome in terms of density.

Thorleifsson et al. [8] carried out a genome-wide association study in Iceland and Sweden and firstly reported tight associations of XFG with 3 single nucleotide polymorphisms (SNPs) of lysyl oxidase-like 1 gene (LOXL1) on 15q24.1. These authors demonstrated one intronic (rs2165241) and two nonsynonymous coding (rs3825942 and rs1048661) SNPs that had significant pathological associations. Similar studies have been conducted in many different species with discrepant results since then [9, 10]. SNPs in the LOXL1 promoter also showed associations with XFS pathogenesis [11, 12]. Our previously reported work confirmed LOXL1 as a susceptibility gene in XFS/XFG among Uygur individuals [13]. With expanded samples and taking into consideration the polymorphisms of multiple SNPs, six SNPs of *LOXL1* were examined, including those in the promoter region (rs4886761, rs4886467, rs4558370, rs4461027, rs16958477, and rs12914489) via direct sequencing.

## 2. Methods

Study subjects: An XFS diagnostic criterion was exfoliation materials detected on the anterior lens capsule or on the pupil margin in at least one eye upon pupil dilation. Individuals with intraocular pressure (IOP) below 21 mmHg and no glaucomatous optic neuropathy were considered XFS cases. XFG diagnosis was based on the above-mentioned exfoliation properties besides the following: (1) IOP ≥ 22 mmHg in at least one eye; (2) glaucomatous alterations of the optic disc, i.e., cup-to-disc ratio above 0.7 in at least one eye or asymmetric cup-to-disc ratio >0.2 or disc rim notching; (3) visual field loss due to glaucoma [14]. Individuals with other factors causing secondary glaucoma, including uveitis, pigment dispersion syndrome, and iridocorneal endothelial syndrome, were excluded. In this study, Kashi and Kuche Uygur individuals above 45 years old were enrolled. The enrolment of control cases was based on the following criteria: (1) no XFS or XFG symptoms; (2) no glaucomatous alterations of the optic disc; (3) normal vision and IOP; (4) no family history of glaucoma; (5) no eye pathology with the exception of mild refractive errors. The participants all had no filial relationships and underwent comprehensive ophthalmic exams.

This study had approval from the Ethics Committee for Human Research of the First Affiliated Hospital of Xinjiang Medical University, China, and followed the Declaration of Helsinki. Each participant provided signed informed consent.

Venous blood specimens (2 to 3 ml) were obtained from individual subjects for DNA extraction with a genomic DNA extraction kit (The Beijing Genomics Institute, China).

All 6 SNPs (rs4886761, rs4886467, rs4558370, rs4461027, rs16958477, and rs12914489) in the LOXL1 promoter were PCR amplified as follows: 11 cycles of 95°C for 2 min, 94°C for 20 s, 65°C for 40 s, and 72°C for 1.5 min; 24 cycles of 94°C for 20 s, 59°C for 30 s, and 72°C for 1.5 min; 72°C for 2 min. The genotypes of these SNPs were also assessed via direct DNA sequencing on an ABI 3730 XL sequencer. GeneMapper v4.1 (Applied Biosystems, USA) was utilized to analyze the data.

### 2.1. Statistical Analysis

SPSS 19.0 (SPSS, USA) was utilized for data analysis. The *χ*2 test was employed for assessing the Hardy–Weinberg equilibrium. Allelic and genotypic frequencies between the cases and the control groups were compared by the *χ*2 test, as well as the haplotype association assessment. *p* < 0.05 indicated statistical significance. Relative risk estimation used odds ratios (OR) and 95% confidence intervals (CI).

## 3. Results

A total of 242 Uygur XFS/G cases including 24 cases of XFG, 14 cases of XFS with high IOP, and 204 cases of XFS without glaucoma and 310 Uygur control patients were enrolled. They were 69.90 ± 8.47 and 68.45 ± 9.94 years old in the case and control groups, respectively (*T*= 1.81, *p* = 0.07). The gender distribution between the patient and control groups was not significantly different (*χ*2= 2.48, *p* = 0.11), with 164 (67.77%) men and 78 (32.23%) women among XFS/G cases, and 190 (61.29%) men and 120 (38.713%) women among controls (Table 1).

The totality of SNPs were firstly submitted to Hardy–Weinberg equilibrium assessment. The results revealed rs12914489 (*p*=0.018) deviated from the HWE in control cases; the remaining SNPs accorded with the HWE (Table 2).

MAF (minor allele frequency) usually refers to the frequency of uncommon alleles in a given population. HapMap plans to take SNPs with MAF >0.05 as the primary research goal. It revealed all MAFs were higher than 0.05, suggesting that the SNPs had statistical significance (Table 3).

Allelic distributions markedly differed between the two groups for all SNPs except for rs12914489 (Table 3). The allele C of rs4886761 had an elevated frequency in XFS/G cases versus controls (*p* < 0.001; OR= 2.204, 95% CI: 1.711–2.838), as well as the allele G of rs4886467 (*p* < 0.001; OR= 1.946, 95% CI: 1.513–2.503) and the allele G of rs4558370 (*p* < 0.001; OR= 0.493, 95% CI: 0.335–0.725). Alleles T of rs4461027 (*p* < 0.001; OR= 2.260 95% CI: 1.773–2.881) and A of rs16958477 (*p* < 0.001; OR= 1.792 95% CI: 1.399–2.297) also had elevated frequencies in XFS/G cases versus controls. The allele G of rs12914489 showed no significant difference between the case and control groups (*p*=0.731; OR= 1.103, 95% CI: 0.631–1.929) (Table 4).

Genotypes TT and CC at rs4886761 had elevated frequencies in XFS/G cases versus controls (OR= 5.655, 95% CI: 3.000–10.660 and OR= 2.241, 95% CI: 1.473–3.408, respectively; both *p* < 0.001), as well as TT and GG at rs4886467 (OR= 4.026, 95% CI: 2.162–7.497 (*p* < 0.001) and OR= 1.631, 95% CI: 1.08–2.463 (*p*=0.02), respectively) and GG at rs4558370 (*p* < 0.001, OR= 0.374, 95% CI: 0.228–0.615). The TT genotype showed no significant difference between the case and control groups (*p*=0.499, OR= 0.611, 95% CI: 0.146–2.555). Genotypes CC and TT at rs4461027 (OR= 5.245, 95% CI: 3.037–9.058 (*p* < 0.001) and OR= 2.210, 95% CI: 1.37–3.564 (*p*=0.001), respectively) and CC and AA at rs16958477 (OR= 3.530, 95% CI: 1.968–6.334 (*p* < 0.001) and OR= 1.740, 95% CI: 1.145–2.646 (*p*=0.009), respectively) had starkly elevated frequencies in XFS/G cases compared with control participants. Genotypes AA and GG at rs12914489 showed similar frequencies in both groups (*p*=0.999 and *p*=0.509, respectively) (Table 5).

**Figure 1:**
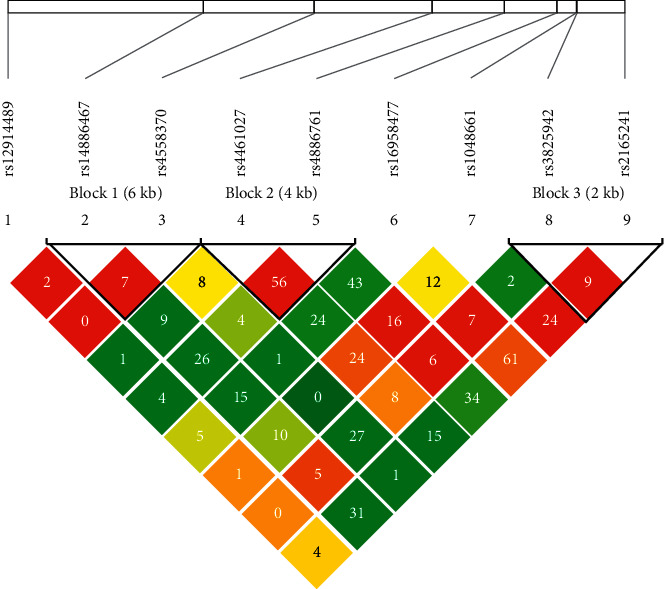
Haploview analysis block diagram of LOXL1.

Next, the 9 SNPs were screened out for haplotype association assessment. Genotyping graphs for the SNPs are depicted (Figure 1).

Haplotypes defined by the six SNPs were analyzed. For the SNPs rs12914489, rs4886467, and rs4558370, four haplotypes were detected. The haplotypes GGT and GTG (OR= 0.474, 95% CI: 0.308–0.730 and OR= 1.909, 95% CI: 1.437–2.535, respectively; both *p* < 0.001) had significant associations with the case group. For rs4461027 and rs4886761, 3 haplotypes were identified, with TC and CT showing significant associations with the case group (OR= 0.427 95% CI: 0.324–0.562 and OR= 2.303, 95% CI: 1.729–3.067, respectively; both *p* < 0.001) (Table 6).

## 4. Discussion

XFS represents an aging disease featuring ECM involvement, with LOXL1 attributed as an essential function in the pathogenetic mechanism [15]. Two common missense mutations in exon 1 and many noncoding variants in the promoter or intron 1 region of LOXL1 have been reported in several XFS/XFG cases globally; therefore, LOXL1 is considered an important effect locus in XFS [8, 16]. According to these findings, besides finding new polymorphisms associated with XFS/G, there were some differences.

The T allele of rs4886761 (OR (95% CI): 2.204 (1.711–2.838)), G of rs4886467 (OR (95% CI): 1.946 (1.513–2.503)), T of rs4461027 (OR (95% CI): 2.26 (1.773–2.881)), A of rs16958477 (OR (95% CI): 1.792 (1.399–2.297)), and G of rs12914489 (OR (95% CI): 1.103 (0.631–1.929)) were risk alleles for the disorder. Meanwhile, the G alleles of rs4558370 (OR (95% CI): 0.493 (0.335–0.725)) were protective.

Genotypes TT and CC for rs4886761 (OR (95% CI): 5.655 (3.000–10.660) and 2.241 (1.473–3.408), respectively), TT and GG for rs4886467 (OR (95% CI): 4.026 (2.162–7.497) and 1.631 (1.08–2.463), respectively), CC and TT for rs4461027 (OR (95% CI): 5.245 (3.037–9.058) and 2.210 (1.37–3.564), respectively), and CC and AA for rs16958477 (OR (95% CI): 3.530 (1.968–6.334) and 1.740 (1.145–2.646), respectively) were risk genotypes for XFS/G. The genotype GG for rs4558370 (OR (95% CI): 0.374 (0.228–0.615)) was protective in this work.

The haplotypes GGT and GTG of rs12914489, rs4886467, and rs4558370; TC and CT of rs4461027 and rs4886761 were found to be significantly associated with XFS/G.

Besides rs12914489 (*p*=0.018) that deviated from the HWE in the control group, the remaining SNPs were in line with the HWE. The frequencies of allele G of rs12914489 (*p*=0.731; OR= 1.103, 95% CI: 0.631–1.929) and genotypes AA and GG at rs12914489 (*p*=0.999 and *p*=0.509, respectively) were comparable in both groups. However, studies have shown that rsl2914489 in the distal region of the LOXL1 promoter is independently related to XFS. It affects gene transcriptional activity by controlling transcriptional binding sites to improve the overall risk of the LOXL1 promoter haplotype [17]. The discrepancy may be caused by an insufficient sample size and/or random or sampling error. Larger studies are warranted to examine rsl2914489's association with XFS/G in Uygur individuals.

LOXL1 was identified as the main genetic risk factor for XFS/G, although causal variants and their respective roles in the pathogenetic mechanisms of XFS still deserve further investigation [18]. Although our previously reported data revealed many genes [19] and SNP polymorphisms pathogenetically critical for XFS/G in Uygur individuals, the pathogenetic roles of these SNPs in XFS/G among Uygur individuals should be accurately examined in future studies determining additional genetic and/or environmental parameters important in XFS/G development.

## Figures and Tables

**Table 1:** The baseline of the two groups.

	Case	Control	*t*	*p*
	*n*= 242	*n*= 310		
Age (years), mean ± SD	69.90 ± 8.47	68.45 ± 9.94	1.81	0.07
Gender (M/F), *n* (%)	164 (67.77%)/78 (32.23%)	190 (61.29%)/120 (38.71%)	2.48	0.11

M: male; F: female.

**Table 2:** The Hardy–Weinberg equilibrium test of these SNPs.

Region	SNP	HWE_case	HWE_control	HWE
Promoter region	rs4886761	0.896	0.664	0.256
Promoter region	rs4886467	0.511	0.888	0.346
Promoter region	rs4558370	0.075	0.136	1
Promoter region	rs4461027	0.29	0.554	0.050
Promoter region	rs16958477	0.192	0.305	0.118
Promoter region	rs12914489	1.000	0.018	0.112

The exception of rs12914489 deviated from the HWE in control cases, and the remaining SNPs all accorded with the HWE.

**Table 3:** The MAF of these SNPs.

SNP	Ref allele	Alt allele	Case MAF	Control MAF	Total MAF
rs4886761	C	T	0.442	0.265	0.342
rs4886467	G	T	0.428	0.277	0.343
rs4558370	G	T	0.085	0.158	0.126
rs4461027	T	C	0.407	0.392	0.480
rs16958477	A	C	0.440	0.305	0.364
rs12914489	G	A	0.050	0.045	0.047

All MAFs were greater than 0.05 besides rs12914489 (almost equal to 0.05), indicating that the SNPs had statistical significance.

**Table 4:** Associations of various alleles with the SNPs.

SNP	XFS/XFG	Control	*χ * ^2^	*p*	OR (95% CI)
rs4886761					
Allele					
C	270	456	38.09	<0.001	2.204 (1.711–2.838)
T	214	164			

rs4886467					
Allele					
G	277	448			
T	207	172	27.22	<0.001	1.946 (1.513–2.503)

rs4558370					
Allele					
G	443	522			
T	41	98	13.29	<0.001	0.493 (0.335–0.725)

rs4461027					
Allele					
T	197	377			
C	287	243	44.01	<0.001	2.26 (1.773–2.881)

rs16958477					
Allele					
A	271	431			
C	213	189	21.47	<0.001	1.792 (1.399–2.297)

rs12914489					
Allele					
G	460	592			
A	24	28	0.119	0.731	1.103 (0.631–1.929)

Allelic distributions markedly differed between the case and control groups for all SNPs with the exception of rs12914489.

**Table 5:** Associations of various genotypes with the SNPs.

SNPs/Genotype	XFS/XFG	Control	*p*	OR (95% CI)
rs4886761				
TT	48	23	＜0.001	5.655 (3.000–10.660)
CT	118	118	＜0.001	2.241 (1.473–3.408)
CC	76	169		

rs4886467				
TT	47	23	＜0.001	4.026 (2.162–7.497)
GT	113	126	0.02	1.631 (1.08–2.463)
GG	82	161		

rs4558370				
TT	4	4	0.499	0.611 (0.146–2.555)
GT	33	90	＜0.001	0.374 (0.228–0.615)
GG	205	216		

rs4461027				
CC	89	50	＜0.001	5.245 (3.037–9.058)
TC	109	143	0.001	2.210 (1.37–3.564)
TT	44	117		

rs16958477				
CC	52	30	＜0.001	3.530 (1.968–6.334)
AC	109	129	0.009	1.740 (1.145–2.646)
AA	81	151		

rs12914489				
AA	0	3	0.999	1.189*e* − 09 (0-inf)
GA	24	22	0.509	1.255 (0.6395–2.462)
GG	218	285		

The various genotypes had significant differences between the case and control groups, with the exception of rs12914489 genotypes.

**Table 6:** The haplotype association analysis between these SNPs.

Haplotype			Case (proportion)	Control (proportion)	*p*-value	OR	95% CI
rs12914489	rs4886467	rs4558370					
G	G	G	186 (0.431)	307 (0.517)	0.048	0.761	0.581–0.997
A	G	G	22 (0.051)	27 (0.045)	0.988	0.995	0.532–1.862
G	G	T	37 (0.086)	95 (0.160)	＜0.001	0.474	0.308–0.730
G	T	G	187 (0.433)	165 (0.278)	＜0.001	1.909	1.437–2.535

	rs4461027	rs4886761					
	T	C	176 (0.407)	366 (0.616)	＜0.001	0.427	0.324–0.562
	C	C	61 (0.141)	75 (0.126)	0.3619	1.200	0.811–1.776
	C	T	195 (0.451)	153 (0.258)	＜0.001	2.303	1.729–3.067

The frequencies of haplotypes were significantly different between the two groups except GGG/AGG of SNPs rs12914489, rs4886467, and rs4558370 and CC of SNPs rs4461027 and rs4886761.

## Data Availability

The (DATA TYPE) data used to support the findings of this study are included within the article. The (DATA TYPE) data used to support the findings of this study are available from the corresponding author upon request if necessary.
